# Supplementary material for: High-Frequency, Low-Intensity Pulsed Electric Field and N-Acetylcysteine Synergistically Protect SH-SY5Y Cells Against Hydrogen Peroxide-Induced Cell Damage In Vitro
Source: Antioxidants (Basel). 2025 Oct 21;14(10):1267. doi: 10.3390/antiox14101267 (PMC12561818; doi:10.3390/antiox14101267)
Supplement: Supplementary file 1 [file antioxidants-14-01267-s001.zip › antioxidants-3918846-supplementary.pdf]

Supplementary Table S1.

The minimal dataset and the full matrix of p-values for all pairwise comparisons.

For Figure 2A

Group numbering	1	2	3	4	5	6
H_2O_2 (µM)	0	100	200	300	400	500
Normalized Viability	99.3	99.6	88.4	75.3	62.9	48.7
	101.1	96.0	92.7	85.1	66.5	55.3
	99.6	94.5	97.1	81.1	65.1	53.5
Average	100.0	96.7	92.7	80.5	64.8	52.5
Standard Deviation	1.0	2.6	4.4	4.9	1.8	3.4

Means comparisons by Tukey test

Groups Compared	p-values
2 1	0.8248
3 1	0.14944
3 2	0.68364
4 1	1.21897E-4
4 2	6.82885E-4
4 3	0.00708
5 1	2.46186E-7
5 2	6.30136E-7
5 3	3.01134E-6
5 4	9.57946E-4
6 1	2.41153E-7

For Figure 2B

Group numbering	1	2	3	4	5	6	7	8
H-LIPEF	-	+	-	+	-	+	-	+
NAC (µM)	0	0	10	10	0	0	10	10
H_2O_2 (µM)	0	0	0	0	500	500	500	500
Normalized Viability	99.5	97.2	100.2	103.3	48.3	65.4	57.3	82.9
	99.3	103.7	100.0	97.6	50.5	59.6	60.7	87.2
	101.2	103.9	99.5	96.2	44.6	60.0	60.7	79.9
Average	100.0	101.6	99.9	99.1	47.8	61.7	59.6	83.3
Standard Deviation	1.1	3.8	0.4	3.8	3.0	3.2	1.9	3.7

Means comparisons by Tukey test

Groups Compared	p-values
2 1	0.99648
3 1	1
3 2	0.99492
4 1	0.99986
4 2	0.95008
4 3	0.99993
5 1	0
5 2	0
5 3	0
5 4	0
6 1	5.03261E-7
6 2	1.38414E-6
6 3	5.01274E-7
6 4	1.65021E-7
6 5	4.76769E-4
7 1	1.83004E-7
7 2	3.56697E-8
7 3	1.04559E-6
7 4	1.35977E-6
7 5	0.00258
7 6	0.9827
8 1	5.7155E-5
8 2	1.83097E-5
8 3	6.14776E-5
8 4	1.16698E-4
8 5	1.53342E-7
8 6	1.88879E-6
8 7	4.83514E-7

For Figure 3B

Group numbering	1	2	3	4
	Ctrl	H_2O_2	H-LIPEF + H_2O_2	H-LIPEF + NAC + H_2O_2
Cells with decreased MMP (%)	25.2	40.9	37.2	28.8
	30.4	41.7	35.8	28.4
	26.9	41.0	32.3	27.6
Average	27.5	41.2	35.1	28.3
Standard Deviation	2.7	0.4	2.5	0.6

Means comparisons by Tukey test

Groups Compared	p-values
2 1	8.72197E-5
3 1	0.00473
3 2	0.01668
4 1	0.9562
4 2	1.32559E-4
4 3	0.00887

For Figure 3C

Group numbering	1	2	3	4
	Ctrl	H_2O_2	H-LIPEF + H_2O_2	H-LIPEF + NAC + H_2O_2
Cleaved PARP / full length PARP (%)	100.0	442.8	366.7	286.6
	100.0	416.9	344.4	293.0
	100.0	441.8	352.4	267.3
Average	100.0	433.8	354.5	282.3
Standard Deviation	0.0	14.7	11.3	13.4

Means comparisons by Tukey test

Groups Compared	p-values
2 1	0
3 1	0
3 2	1.29427E-4
4 1	1.044E-7
4 2	8.34325E-7
4 3	2.54897E-4

For Figure 4B

Group numbering	1	2	3	4
	Ctrl	H_2O_2	H-LIPEF + H_2O_2	H-LIPEF + NAC + H_2O_2
Superoxide level (Fold relative to control)	1.00	1.67	1.37	1.14
	1.00	1.60	1.52	1.16
	1.00	1.71	1.36	1.00
Average	1.00	1.66	1.42	1.10
Standard Deviation	0.00	0.05	0.09	0.08

Means comparisons by Tukey test

Groups Compared	p-values
2 1	1.11999E-5
3 1	3.31161E-4
3 2	0.01042
4 1	0.344
4 2	3.88525E-5
4 3	0.00211

For Figure 5A

Group numbering	1	2	3	4
	Ctrl	H_2O_2	H-LIPEF + H_2O_2	H-LIPEF + NAC + H_2O_2
p-Akt / GAPDH (%)	100.0	54.3	63.5	107.8
	100.0	44.7	77.5	102.1
	100.0	39.2	60.6	106.3
Average	100.0	46.1	67.2	105.4
Standard Deviation	0.0	7.6	9.0	3.0

Means comparisons by Tukey test

Groups Compared	p-values
2 1	2.16825E-5
3 1	7.79857E-4
3 2	0.01205
4 1	0.7081
4 2	1.04798E-5
4 3	2.71904E-4

For Figure 5B

Group numbering	1	2	3	4
	Ctrl	H_2O_2	H-LIPEF + H_2O_2	H-LIPEF + NAC + H_2O_2
Nrf2 / GAPDH (%)	100.0	53.4	67.1	93.4
	100.0	48.4	64.8	91.1
	100.0	52.1	73.3	85.1
Average	100.0	51.3	68.4	89.9
Standard Deviation	0.0	2.6	4.4	4.3

Means comparisons by Tukey test

Groups Compared	p-values
2 1	3.21486E-7
3 1	1.2752E-5
3 2	0.00107
4 1	0.02421
4 2	2.67686E-6
4 3	2.22937E-4

For Figure 5C

Group numbering	1	2	3	4
	Ctrl	H_2O_2	H-LIPEF + H_2O_2	H-LIPEF + NAC + H_2O_2
SOD2 / GAPDH (%)	100.0	98.0	118.7	136.1
	100.0	82.3	118.4	147.8
	100.0	91.1	131.7	148.5
Average	100.0	90.5	122.9	144.2
Standard Deviation	0.0	7.8	7.6	7.0

Means comparisons by Tukey test

Groups Compared	p-values
2 1	0.33926
3 1	0.01073
3 2	0.00126
4 1	1.49644E-4
4 2	3.56421E-5
4 3	0.01654

For Figure 5D

Group numbering	1	2	3	4	5
	Ctrl	H_2O_2	NAC + H-LIPEF + H_2O_2	LY294002 + H_2O_2	LY294002 + NAC + H-LIPEF + H_2O_2
Normalized Viability	99.7	56.1	81.2	49.5	58.7
	99.1	54.2	82.7	49.7	56.1
	101.2	51.4	77.8	47.7	57.9
Average	100.0	53.9	80.5	49.0	57.6
Standard Deviation	1.1	2.4	2.5	1.1	1.3

Means comparisons by Tukey test

Groups Compared	p-values
2 1	0
3 1	1.29219E-6
3 2	0
4 1	0
4 2	0.04158
4 3	0
5 1	0
5 2	0.16553
5 3	6.69701E-8
5 4	0.00108

For Figure 6B

Group numbering	1	2	3	4
	Ctrl	H_2O_2	H-LIPEF + H_2O_2	H-LIPEF + NAC + H_2O_2
Intracellular H_2O_2 level (Fold relative to control)	1.00	1.97	1.73	1.33
	1.00	2.08	1.80	1.56
	1.00	2.09	1.68	1.30
Average	1.00	2.05	1.74	1.40
Standard Deviation	0.00	0.07	0.06	0.14

Means comparisons by Tukey test

Groups Compared	p-values
2 1	1.46804E-6
3 1	2.33132E-5
3 2	0.00849
4 1	0.00187
4 2	5.93164E-5
4 3	0.00491

For Figure 7B

Group numbering	1	2	3	4
	Ctrl	H_2O_2	H-LIPEF + H_2O_2	H-LIPEF + NAC + H_2O_2
Avidin-Alexa 488 / DAPI (Fold relative to control)	1.14	4.23	1.92	1.02
	1.02	3.57	2.36	1.34
	0.84	3.66	2.61	1.17
Average	1.00	3.82	2.30	1.18
Standard Deviation	0.15	0.36	0.35	0.16

Means comparisons by Tukey test

Groups Compared	p-values
2 1	6.62982E-6
3 1	0.00179
3 2	6.09354E-4
4 1	0.85621
4 2	1.0927E-5
4 3	0.00451

For Figure 7C

Group numbering	1	2	3	4
	Ctrl	H_2O_2	H-LIPEF + H_2O_2	H-LIPEF + NAC + H_2O_2
MTH1 / GAPDH (%)	100.00	53.32	66.39	82.51
	100.00	42.37	55.78	89.73
	100.00	53.28	69.22	92.53
Average	100.0	49.7	63.8	88.3
Standard Deviation	0.0	6.3	7.1	5.2

Means comparisons by Tukey test

Groups Compared	p-values
2 1	1.45686E-5
3 1	1.67611E-4
3 2	0.04978
4 1	0.10713
4 2	1.05391E-4
4 3	0.00242
